# Robotic Surgery: Anaesthesiologist’s Contemplation

**DOI:** 10.21315/mjms2020.27.3.15

**Published:** 2020-06-30

**Authors:** Arpita Kapur, Vinay Kapur

**Affiliations:** 1 Department of Anaesthesiology, Civil Hospital Sector-6, Panchkula, Haryana, India; 2 Department of Medicine, H S Judge Institute of Dental Sciences, Panjab University, Chandigarh (UT), India

**Keywords:** robotic surgery, anaesthesia complications, pneumoperitoneum, patient positioning

## Abstract

Technological advances in the field of surgery and medicine have increased the demand for minimally invasive surgery manifold. Robot assisted surgery is gaining popularity, overcoming the flaws of laparoscopic techniques; with improved surgical precision. The conservative nature of anaesthesia care has to face the challenges with respect to patient positioning, bulkiness of the operating system and being positioned far and away from the patient. Anaesthesiologist’s commitment to be the ‘best man’ for the patient during the perioperative period mandates him to familiarise with these challenges of robot assisted surgical system and provide best possible anaesthetic care and ensure patient safety. In this article, a systematic review of the development of surgical robots and the consideration of unique anaesthetic concerns thereof have been undertaken as any new technology is known to be accompanied by its risks and technical perplexity.

## Introduction

A robot is technically defined as a ‘powered, computer controlled manipulator with artificial sensing that can be reprogrammed to move and position tools to carry out a wide range of tasks’ (1). The present day medical robotic systems originated in the United States Department of Defence to enable surgeons to have access to treat exsanguinating soldiers in the battlefield from safe distance; thus came the telerobotic surgery. In 1985, NASA (National Air and Space Administration) in collaboration with Stanford Research Institute developed a telemanipulator (2). The first robotic assisted surgery was performed in 1985 to take a neurosurgical biopsy (3). Today’s robotic systems used in surgery are basically computer assisted devices and more precisely computer enhanced telemanipulators.

## Robotic Systems

The concept of telerobotic surgery eventually merged with laparoscopic surgery and on July 11, 2000 (4) developed into two Food and Drug Administration (FDA) approved telesurgical devices: the da Vinci robotic system and the Zeus robotic surgical system.

**Figure 1:**
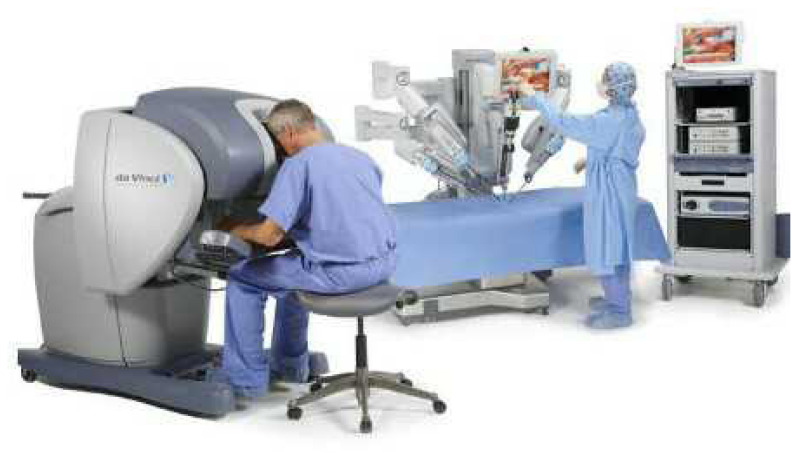
Robotic surgery set up

The da Vinci system (Figure 1) mimes a human wrist and comprises of three distinct parts (5):

- A control console: the surgeon is seated here to visualise and control the robot from a distant location. The other capabilities of console include video system and robotic arms adjustment, comfortable ergonomic adjustment
- An optical three dimensional vision tower: contains computer equipment utilised to integrate the left and right optical channels into stereoscopic vision for the operating room team
- The surgical cart/robot: has four arms being controlled by the surgeon in the console. This is quite bulky. The robot enables more ergonomic, anatomic control of surgical instruments allowing seven degrees of freedom

The da Vinci Si HD surgical system has a dual console to support training, up to 10′ magnification, an ‘endowrist’ that provides the dexterity of human hand and ‘intuitive motion’ technology which provides eye-hand-instrument alignment equivalent to the experience of open surgery.

## Limitations of Robotic Surgery

- Large operating room space required for accommodating bulky equipments
- Robot malfunction or crash down in the middle of the surgery
- The motion of robotic arms may lead to collision with assistants, its own arms and the patient
- Invasion of anaesthesia work space by robot making the patient inaccessible intraoperatively
- Reposition of the patient in the middle of the surgery is impractical once the robot is fixed for surgery
- Lack of tactile feedback from the instruments forces the surgeon to rely only on visual cues to regulate the force and pressure on the tissues to avoid any damage
- Increased surgical operating times as the surgeons and the team climb up the learning curve
- Large initial cost of purchasing and setting up the robotic systems along with increased operating room set up time and surgical time adds considerably to the finances involved
- Latent time required to send the electrical signal from hand motion to actual visualisation of hand motion on a remote screen is a major drawback that needs to be addressed. This is likely to interfere with surgical accuracy and safety. Delays less than 200 ms can be compensated by humans (7)
- Incompatibility with imaging equipments

## Enumeration of Robotic Assisted Surgeries

During the past decade, robot assisted surgeries are being undertaken for a wide variety of operations. The da Vinci system was first of all utilised for cardiothoracic surgeries. Today, this system is being utilised for gastrointestinal surgeries, urologic surgeries, neurosurgeries, gynaecological surgeries as enumerated below:

- Cardiothoracic surgeries: thymectomy, oesophagectomy, lobectomy, resection of oesophageal and mediastinal masses, septal defect closures, valvular repairs, pericardial window, of pacemaker lead placement, endoscopic coronary artery bypass grafting etc. (4, 8)
- Gastrointestinal surgeries: gastrectomy, gastric bypass, splenectomy, cholecystectomy, bowel resection, pyloroplasty, adrenalectomy, pancreatoduodenectomy etc. (9)
- Urological surgeries: radical prostatectomy, cystectomy with urinary diversion, pyeloplasty, pelvic lymph adenectomies, partial and total nephrectomy etc. (10)
- Gynaecological surgeries: benign hysterectomy, sacrocolpopexy, myomectomy, fallopian tube anastomosis etc. (11)
- Other areas: transoral robotic surgery including radical tonsillectomy, tongue base resection, supraglottic laryngectomy; retinal surgery, total hip and knee arthroplasties etc. The modern anaesthesiologist is expected to keep abreast of these changes in surgical field and consider their impact on anaesthetic plan and patient safety.

## Anaesthesiologist’s Contemplation

A general forethought in robot assisted surgeries can be summarised as below:

### Pre-Anaesthetic Check-Up

A detailed history and physical examination is carried out to evaluate the whether the patient is capable of combating the challenges associated with a prolonged surgery in specified position. Pulmonary dysfunction poses a high risk for such procedures as it results in high peak airway pressures to overcome the raised intra-abdominal pressure due to pneumoperitoneum and steep trendelenburg position. Obesity poses a risk of difficult intubation, higher risk of coronary artery disease, pulmonary dysfunction, diabetes and prolonged surgical time (12). Arterial oxygenation is impaired in such patients under general anaesthesia in trendelenburg position. Pneumoperitoneum adding to the impairment. Any pre-existing nerve injury must be documented in detail.

### Pre-operative Instructions

Patient is asked to stop oral intake from midnight preceding the surgery. Benzodiazepine and an H_2_ antagonist are given on the preceding night and in the morning of surgery with sip of water to prevent anxiety and the risk of aspiration pneumonitis. Currently, clear carbohydrate drinks containing maltodextrins, complex carbohydrates that empty out fast from the stomach are recommended as oral pre-loads. The enhanced recovery after surgery (ERAS) programme recommends first dose of oral pre-load of 800 mL for 12 h before surgery followed by the second dose of 400 mL for 2 h–3 h before surgery for maximum benefit. This avoids dehydration before surgery, allows safe general anaesthesia with empty stomach without increased risk of aspiration. It also gives a large carbohydrate load to keep the patient in a metabolically fed state before surgery thus reducing insulin resistance and catabolism and preserving muscle function as measured by grip strength (13). The type-2 diabetic patients can be given a pre-load along with their usual diabetic medication. Pre-load has shown significant effect on reducing complications and improving wellbeing apart from reducing the intravenous fluids requirement.

### Intra-operative Preparation and Monitoring

After confirming the nil per os or nothing by mouth (NPO) status of the patient, multipara monitors are applied and all the relevant parameters are monitored throughout the surgery. Long surgeries, cold intravenous fluids, respiratory gases and CO_2_ insufflation can lead to hypothermia thus making temperature monitoring important along with use of warm fluids and warm humidified gases (14). Two intravenous cannulae with extension tubings must be secured. Antisialagogue administration intravenously is mandatory to prevent aspiration pneumonitis in specific position. Central venous catheter may be required in procedures involving major fluid shift in order to measure central venous pressure. Arterial line may be essential for certain surgeries. Deep vein thrombosis prophylaxis should strictly be followed. A trial of the final patient position should be done to anticipate any strain, on intravenous tubings, monitoring cables and anaesthesia circuit. Defibrillator pad/external transthoracic defibrillation, transesophageal echocardiography (TEE), precordial stethoscope in paediatric patients should be in place before the robot is docked. Tube position can be confirmed with a fiberoptic bronchoscope.

### Induction of Anaesthesia

A suitable anaesthetic agent consistent with the patient’s pathophysiological condition may be used. Oxygen is used along with inhalational agent preferably Sevoflurane along with fentanyl for uniform analgesia. Complete muscle relaxation is essential for enabling introduction of surgical equipments, creation of pneumoperitoneum that facilitates optimal working space and visualisation. Neuromuscular blockade can be achieved with atracurium or rocuronium. Movement of the patient while the robotic instruments are docked can result in tearing or puncturing of organs and vasculature with calamitous consequences (15). Epidural catheter placement helps in intra- and post-operative pain relief in addition to reducing the gut volume. The patient should be safely strapped to the table to prevent sliding down after positioning.

### Patient Positioning

This is the most critical part of robotic surgery that needs to be carried out under the direct supervision of surgeon and anaesthesiologist. Surgeries of pelvic area are usually done in lithotomy and steep trendelenburg position; those in upper abdomen and diaphragm are performed in reverse trendelenburg supine position. Chest surgery is performed in the lateral position with variations of trendelenburg or reverse trendelenburg according to surgical site. Laparoscopic surgeries require specified position in order to take advantage of gravitational effect that allows movement of the obstructing organs away from the field (5). Pressure points must be protected at the time of positioning because of limited access and prolonged duration of surgery. Cushioned stirrups should be used in lithotomy. It is advisable to tuck the arms and hands by the side of the patient after being generously padded to avoid tissue and nerve impingement and kinking of tubings. In such position the patient should be well secured and strapped to the operating table to avoid slipping. Klauschie et al. (16) demonstrated the use of an anti-skid foam material for secure patient positioning. Any suspected perioperative nerve injury should be immediately worked up with electromyography and nerve conduction studies to provide clinical information. Bilateral eye patches must be applied to avoid corneal abrasions. Robotic arms, cameras and light sources should be carefully monitored to avoid injury to the patient. Pneumoperitoneum along with acute patient positioning can cause migration of endotracheal tube into the right bronchus. Tube position must be reconfirmed before the robot is docked. It is advisable to put a nasogastric tube to decompress the stomach as the reflux from the stomach may cause oral ulceration and conjunctival burns and patient’s face kept visible throughout the surgery (17). Steep trendelenburg position for a prolonged period of time can result in facial, upper airway and brain oedema, increase in cerebral blood flow and intracranial pressure (10). Maintenance of normocarbia is essential for preserving cerebrovascular homeostasis. Increase in inspired oxygen concentration may be required to overcome any compromise in oxygenation due to steep trendelenberg position. Molloy’s study showed that even under anaesthesia, cerebrovascular and ophthalmic circulatory auto-regulation do not prevent increase in the intraocular pressure (IOP) (18). IOP can increase by 13 mmHg or higher than the baseline. Increased arterial CO_2_ can lead to choroidal vasodilatation which mandates positive pressure ventilation, hemodynamic maintenance, ventilation strategy and fluid management.

### Pneumoperitoneum

The combination of pneumoperitoneum and steep trendelenburg position cause various cardiovascular perturbations. There is two to three fold rise in left ventricular filling pressure while cardiac output may decrease. There is increase in systemic vascular resistance and mean arterial pressure; however, there is reduction in renal, splanchnic and portal blood flow. Levels of vasopressin increase due to activation of renin angiotensin system (19). Pneumoperitoneum can result in subcutaneous emphysema, pneumothorax, pneumomediastinum and in a worst case, gas embolism. Clinically, gas embolism can manifest as a sudden rise followed by a rapid fall in end-tidal CO_2_, hypotension, tachycardia, diminished breath sounds in a specific lung area, cyanosis and a typical mill-wheel murmur. Anaesthesiologists need to be vigilant in detecting gas embolism as it may require discontinuation of N_2_O and restriction of intra-abdominal pressure and CO_2_ insufflation. Although hyperventilation is the solution to hypercarbia and respiratory acidosis but this is restricted here during to the steep trendelenburg position. Use of pressure controlled ventilation delivering a larger tidal volume for same inspiratory pressure can be useful in difficult to ventilate patients. Increased peak and plateau airway pressures while decrease in pulmonary compliance and vital capacity, may lead to ventilation-perfusion mismatch. Positive end expiratory pressure (PEEP) can help decrease atelectasis (20). PEEP improves oxygenation and lung mechanics, impedes venous return to heart thus decreasing cardiac output; however, these effects are overcome by the steep trendelenberg position.

### Patient Access

Access to the patient during the surgery becomes the anaesthesiologist’s primary concern (21). In some surgeries, patient’s airway has to be at a distance from the anaesthesiologist and his workstation/monitors. During upper abdominal and thoracic surgeries, the operation room table needs to be rotated 180° away from the anaesthesiologist with robot positioned at the patient’s head end. In mediastinal procedures the table is rotated 90° away. In such cases access to patient’s airway in practically impossible. The access constraint is particularly important in an emergency situation. Cardiopulmonary resuscitation is difficult with the robot docked. Position of the operating table cannot be disturbed unless the robot is disengaged and removed. Although a multistep process, if done by well trained staff, removal of the robot can be completed in less than one minute. Surgical instruments and camera are removed from the robot arms, trocars are removed from the arms and the entire robot apparatus is the backed away from the patient. Endotracheal tube must be secured firmly due to the positioning of the patient and movement of the robot arms anticipating limited access during the surgery.

### Maintenance of Anaesthesia

Anaesthesia is maintained with oxygen, air, sevoflurane and remifentanil. Neuromuscular blockade can be achieved with top ups of atracurium or rocuronium. Remifentanil mitigates the nociceptive stress response, provides adequate intraoperative analgesia and suppresses respiration also allowing intubation without muscle relaxant (22). Long surgical times, pneumoperitoneum and steep trendelenburg position can make the fluid management a complex issue. Blood volume replacement monitoring is required for occult blood loss. As in case of vesicourethral anastomosis, excessive urine output can obscure the operating field and occlude the judgement regarding fluid requirement. Excessive fluid infusion can lead to facial, laryngeal and pharyngeal oedema complicating the extubation. Restricting the amount of CO_2_ insufflation causing venous congestion in upper extremity; can help in preventing facial and airway oedema. Intra-operative haematocrit can be a guide for fluid management in prolonged surgeries. It is recommended to keep the pre and intra-operative fluids to < 2 L of crystalloid (23). Crystalloids have a short lived existence in the intravascular compartment. Diuresis can be induced with frusemide or mannitol. This helps to conserve renal function, promotes flow of urine to flush out and maintain the patency of urinary tract and guards against cerebral oedema in trendelenburg position. Protection against cerebral oedema can be achieved with fluid restriction, normocarbia, minimum insufflation pressure, use of diuretic agent.

### Reversal of Anaesthesia

The anaesthetic agents must be discontinued early nearly around the time the robot is removed. Cerebral oedema and raised intracranial tension in prolonged surgery in head down position can delay cognitive recovery. This complication can be reduced as the surgeon progresses along the learning curve. Presence of periorbital oedema should prompt the anaesthesiologist to expect concomitant airway oedema. Laryngeal oedema can lead to stridor following extubation of trachea (24). Anaesthesiologist must remain vigilant against aspiration post extubation and also remain watchful against compartment syndrome in calves after prolonged lithotomy. Continuous positive airway pressure (CPAP) and bilevel positive airway pressure (BiPAP) can be continued into the post-operative period to prevent atelectasis (25).

### Post-Operative Pain Relief

The incidence of severe post-operative pain is less in comparison to open procedures. Simple analgesics and opioid boluses generally suffice. Epidural analgesia is indicated in surgeries requiring perioperative sympathetic blockade in addition to pain relief. Multimodal pain management and appropriate post operative nausea and vomiting prophylaxis are likely to reduce the hospital stay and improve patient satisfaction (25).

## Team Dynamics and Good Communication

A surgical programme cannot be expected to be successful merely by ensuring the availability of robotic surgery setup. Teamwork is mandatory for achieving success. Anaesthesiologists must be prepared to handle the consequences of long duration of surgery, stressed surgeons and deep learning curve. Robotic surgery simulation programmes provide safe opportunity to improve outcomes, reduce morbidity while enhancing team spirit. Above all, good communication among the team members including surgeons, anaesthesiologists, nurses and technical staff is a key to a safe and efficient environment. Concise communication spoken with distinctive speech in a controlled tone of voice with mutual respect can do wonders.

## Conclusion

Robotic surgery is picking up pace with the times and has grown into a subspecialty of its own. Anaesthesiologists need to get well acquainted with this novel surgical trend, keeping in mind its limitations. Anaesthetic techniques and the method of delivery specific for robotic-assisted surgery must be practiced to improve patient care and thus the surgical outcomes. The advantages of robotic surgery over conventional surgery including improved surgical precision, better visualisation and more ergonomic instrument control are enabling faster surgical curves for surgeons. Teamwork and communication among hospital administration, surgeons, anaesthesiologists, nursing staff and technical staff in addition to patience for accommodating the learning curve; is essential to handle the associated complications, and provide safe patient care.
